# Better survival of patients with hepatitis B virus-related hepatocellular carcinoma in South Korea: Changes in 16-years cohorts

**DOI:** 10.1371/journal.pone.0265668

**Published:** 2022-03-24

**Authors:** Sang Il Choi, Yuri Cho, Moran Ki, Bo Hyun Kim, In Joon Lee, Tae Hyun Kim, Seong Hoon Kim, Young Hwan Koh, Hyun Beom Kim, Eun Kyung Hong, Chang-Min Kim, Joong-Won Park

**Affiliations:** 1 Center for Liver and Pancreatobiliary Cancer, National Cancer Center, Goyang, Republic of Korea; 2 Graduate School of Cancer Science and Policy, National Cancer Center, Goyang, Republic of Korea; 3 Department of Radiology, National Cancer Center, Goyang, Republic of Korea; 4 Center for Proton Therapy, National Cancer Center, Goyang, Republic of Korea; Middle East Liver Diseases (MELD) Center, Tehran, Iran, ISLAMIC REPUBLIC OF IRAN

## Abstract

**Aims:**

The incidence and mortality of hepatocellular carcinoma (HCC) have decreased over time in South Korea, where hepatitis B virus (HBV) in endemic. This study investigated the changes in the characteristics and clinical outcomes of HCC patients in Korea.

**Methods:**

Patients initially diagnosed with HCC and treated at the National Cancer Center, Korea between 2000 and 2015 (n = 4,291) were followed up until February 2017. Differences in patient characteristics and outcomes were compared between chronological cohorts: cohort A (2000–2004, n = 1,157) *vs*. B (2005–2009, n = 1,678) *vs*. C (2010–2015, n = 1,456).

**Results:**

The median age of the patient cohort was 57 years (range, 13–98 years), and male predominance was noted (81.6%). HBV infection was the most common etiology (74.8%). The proportion of patients diagnosed with good liver function and small tumors (<2 cm) increased significantly over time: 74.6%, 79.9%, and 87.4% for Child–Pugh class A (*p*<0.001) and 8.0%, 8.5%, and 12.0% for modified UICC stage I (*p*<0.001) in cohorts A, B, and C, respectively. Median overall survival improved significantly over time: 14.4 months (95% confidence interval [CI], 12.0–16.8 months), 22.9 months (95% CI, 20.3–25.5 months), and 53.6 months (95% CI, 45.7–61.5 months) in cohorts A, B, and C, respectively. HBV-related patients showed significantly improved survival (12.7 *vs*. 20.4 *vs*. 64.5 months, *p*<0.001) associated with the use of antiviral treatments (adjusted hazard ratio, 0.72; 95% CI, 0.64–0.80).

**Conclusions:**

The survival of patients with HCC, especially HBV-related HCC, has improved significantly over time in Korea.

## Introduction

Liver cancer is the 5th most common malignancy in males and 9th in females, accounting for more than 700,000 annual deaths globally [1]. Hepatocellular carcinoma (HCC) accounts for 85% to 90% of all liver cancers. In South Korea, where hepatitis B virus (HBV) infection is endemic, HCC is the 5th most common malignancy in both sexes, is the 2nd most common cause of cancer-related death [2]. The incidence and mortality of HCC is reported to be declining in the last 20 years [3, 4]. This is believed to be associated with the introduction of the National Cancer Screening Program (NCSP), which facilitates early detection and treatment [5, 6], establishment of evidence-based treatment guidelines [7, 8], and development of new therapeutic modality [9]. Especially for patients with HBV-related HCC, the advent of potent antiviral agents has substantially reduced liver-related morbidity and mortality [10].

We previously reported the clinical characteristics and outcomes of a cohort that included patients with HCC who were prospectively enrolled at the National Cancer Center (NCC) in Korea [11, 12]. In this study, we further evaluated the chronological evolution of clinical characteristics and treatment outcomes of patients with HCC by comparing cohorts that included patients who were diagnosed in different time periods during 2000–2015 in the NCC. The aim of our study was to investigate the changes in the characteristics and clinical outcomes of patients with HCC in Korea between 2000 and 2015, and also examine the effect of antiviral treatment against HBV infection on the stage of HBV-related HCC at diagnosis and overall mortality as compared to the outcomes of patients with non-HCC-related HCC.

## Materials and methods

### Patients

**Fig 1:**
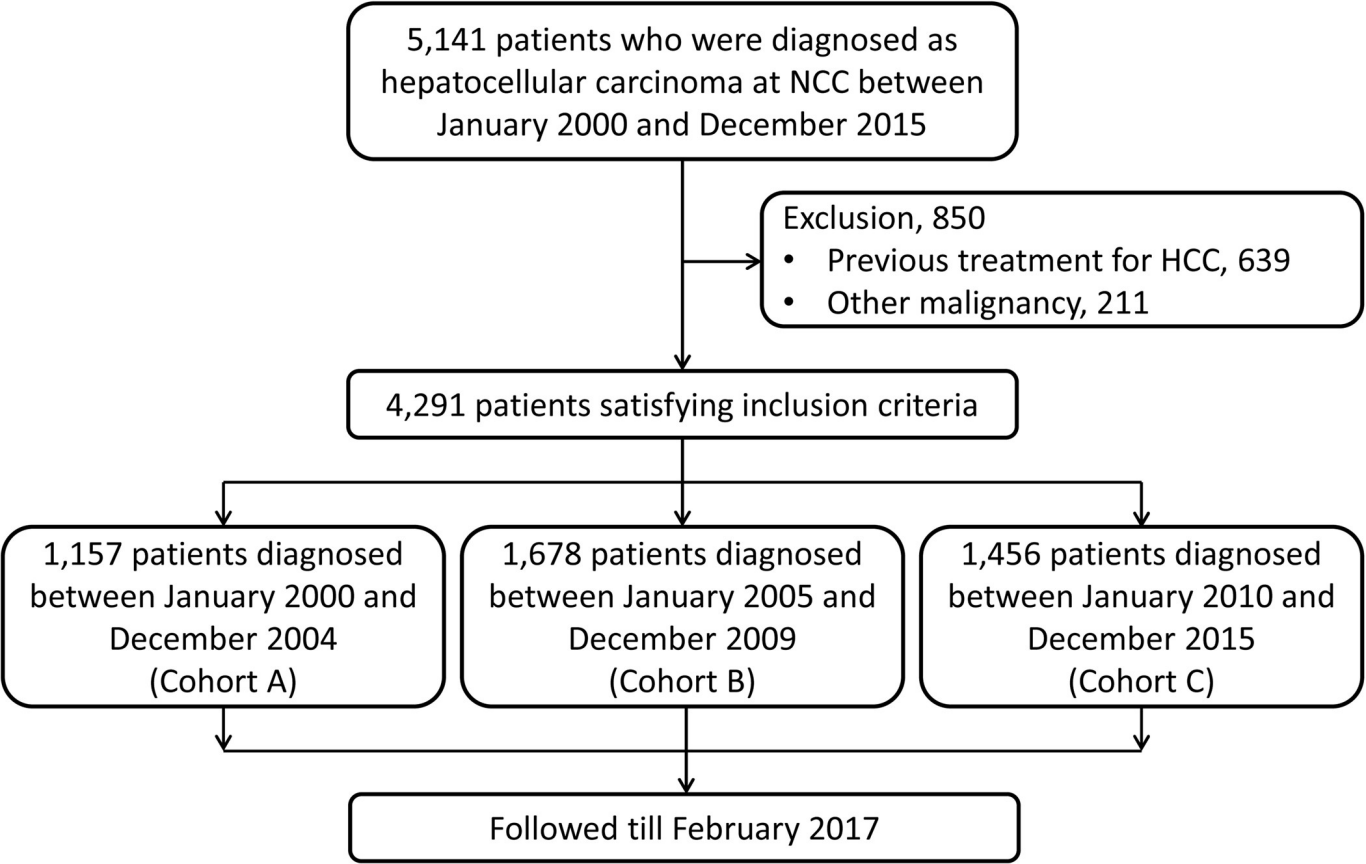
Study design.

Between January 2000 and December 2015, a total of 5,141 patients visited the NCC in South Korea with the impression of primary liver cancer and were enrolled in this prospective cohort of HCC in the NCC. Patients with a previous history of other invasive malignancies and those who had previous treatment for already diagnosed HCC at an outside hospital were excluded from the analysis (Fig 1).

Patients were divided into three subgroups according to the year when patients were first diagnosed with HCC: cohort A for those who were diagnosed with HCC from 2000 to 2004; cohort B, from 2005 to 2009; and cohort C, from 2010 to 2015 (Fig 1). Patients were followed up until February 2017.

Performance status (PS) was evaluated according to the grading system suggested by the Eastern Cooperative Oncology Group (ECOG) based on patient reports and physical examination. Other clinical information such as etiology, baseline liver function, tumor size, number of tumor nodules, presence of portal vein invasion and extrahepatic metastasis, tumor markers such as alpha-fetoprotein (AFP) and protein induced by vitamin K antagonist-II (PIVKA-II), initial treatment modality, use of antiviral agents for underlying chronic viral hepatitis, and survival were collected prospectively.

This study was conducted in accordance with the provisions of the Declaration of Helsinki and was approved by the Institutional Review Board of the NCC, South Korea (NCC 2017–0119). All participants in the study cohort provided written informed consent.

### Diagnosis and treatment

Patients were diagnosed according to the practice guidelines proposed by the Korea Liver Cancer Study Group and NCC, Korea [7, 13], and were further staged according to the modified Union for International Cancer Control (mUICC) staging [14], and Barcelona Clinic for Liver Cancer (BCLC) staging system [15, 16]. Liver function was evaluated using the Child–Pugh score and the model for end-stage liver disease (MELD) scoring system [17, 18].

Tumors were considered to be well-defined if the margin of the tumor nodule was clearly delineated from the non-tumor liver parenchyma on liver dynamic computed tomography (CT) or liver gadoxetic acid (Primovist)-enhanced magnetic resonance imaging (MRI). Tumor number was defined as the number of intrahepatic tumor nodules. Tumor size was measured for the largest measurable tumor nodule on CT or MRI. The clinical decision as to which treatment would be appropriate for each patient was made at the discretion of each physician according to the patient’s clinical situation in reference to practice guidelines [7].

### Statistical analysis

The differences in baseline characteristics and clinical outcomes were compared between predefined subgroups. Continuous variables were categorized for comparison. Categorized variables and categorical variables are shown as frequencies with percentages. The differences in each variable between subgroups were tested using the Pearson’s chi-square test. Survival analysis was performed using the Kaplan-Meier method. Differences in overall survival (OS) were evaluated using the log-rank test. The Cox proportional hazard model was used to identify factors associated with patient survival. Variables for the final multivariable model were selected among the variables with *p*<0.05, in univariate analysis while considering the possibility of multicollinearity. Hazard ratios (HRs) for mortality are shown with 95% confidence interval (CI). Statistical significance was set at *p*<0.05. All statistical analyses were performed using SPSS version 21 (IBM Corp., Armonk, NY, USA).

## Results

### Baseline characteristics

The study finally included 4,291 patients (Fig 1). Detailed characteristics are shown in Table 1. The most frequent etiology was HBV infection, which accounted for 74.8% of the entire cohort. However, the proportion of HBV-related HCC decreased as the cohort moved from A to C. In cohorts A, B, and C, the proportion of HCC with viral etiology (HBV-related or hepatitis C virus [HCV]-related HCC) significantly decreased from, while alcohol-related HCC increased (S1 Fig).

**Table 1:** Baseline characteristics.

	Total (n = 4,291)	Cohort A (n = 1,157)	Cohort B (n = 1,678)	Cohort C (n = 1,456)	*p-value* ^§^
**Age**					
Mean (SD)	57.1 (10.5)	56.0 (10.3)	56.6 (10.5)	58.5 (10.6)	<0.001**
<50, n (%)	1,036 (24.1%)	309 (26.7%)	443 (26.4%)	284 (19.5%)	<0.001
≥50, n (%)	3,255 (75.9%)	848 (73.3%)	1,235 (73.6%)	1,172 (80.5%)	
**Sex, n (%)**					
Male	3,502 (81.6%)	932 (80.6%)	1,398 (83.3%)	1,172 (80.5%)	0.070
Female	789 (18.4%)	225 (19.4%)	280 (16.7%)	284 (19.5%)	
**Etiology, n (%)**					
HBV	3,211 (74.8%)	886 (76.6%)	1,249 (74.4%)	1,076 (74.0%)	<0.001
HCV	375 (8.7%)	110 (9.5%)	154 (9.2%)	111 (7.6%)	
Alcohol	318 (7.4%)	64 (5.5%)	122 (7.3%)	132 (9.1%)	
HBV+HCV	18 (0.4%)	1 (0.1%)	1 (0.1%)	16 (1.1%)	
Other	368 (8.6%)	96 (8.3%)	152 (9.1%)	120 (8.2%)	
**Nucleos(t)ide analogue treatment, n (%)**	1,439	100	424	915	<0.001
Lamivudine	261 (18.1%)	51 (51%)	190 (44.8%)	20 (2.2%)	
Adefovir	110 (7.6%)	16 (16%)	84 (19.8%)	10 (1.1%)	
Entecavir	582 (40.4%)	24 (24%)	128 (30.2%)	430 (47.0%)	
Tenofovir disoproxil fumarate	435 (30.2%)	4 (4%)	21 (5.0%)	410 (44.8%)	
Clevudine or telbivudine	24 (1.7%)	5 (5%)	1 (0.2%)	18 (2.0%)	
Entecavir+tenofovir disoproxil fumarate	27 (1.9%)	0 (0%)	0 (0%)	27 (3.0%)	
**HCV treatment, n (%)**	69 (18.4%)	10 (9.1%)	32 (20.8%)	27 (24.3%)	<0.001
IFN-based regimen	49 (71.0%)	10 (100%)	28 (87.5%)	11 (40.7%)	
DAA	20 (29.0%)	0 (0%)	4 (12.5%)	16 (59.3%)	
SVR	44 (63.8%)	6 (60%)	15 (46.9%)	23 (85.2%)	0.012
**ECOG PS, n (%)**					
0	2,230 (54.3%)	385 (33.3%)	816 (48.6%)	1,129 (77.5%)	<0.001
1	1,864 (43.4%)	750 (64.8%)	821 (48.9%)	293 (20.1%)	
2	88 (2.1%)	22 (1.9%)	38 (2.3%)	28 (1.9%)	
3	9 (0.2%)	0 (0.0%)	3 (0.2%)	6 (0.4%)	
**Child–Pugh class** ^*^ **, n (%)**					
A	3,408 (81.1%)	800 (74.6%)	1,340 (79.9%)	1,268 (87.4%)	<0.001
B	687 (16.4%)	224 (22.9%)	296 (17.6%)	167 (11.5%)	
C	106 (2.5%)	48 (4.5%)	42 (2.5%)	16 (1.1%)	
**mUICC stage, n (%)**					
I	410 (9.6%)	92 (8.0%)	143 (8.5%)	175 (12.0%)	<0.001
II	1,304 (30.4%)	277 (23.9%)	503 (30.0%)	524 (36.0%)	
III	1,295 (30.2%)	411 (35.5%)	516 (30.8%)	368 (25.3%)	
IVa	828 (19.3%)	222 (19.2%)	298 (17.8%)	308 (21.2%)	
IVb	454 (10.6%)	155 (13.4%)	218 (13.0%)	81 (5.6%)	
**BCLC stage** ^*^ **, n (%)**					
0	258 (6.1%)	29 (2.7%)	87 (5.2%)	142 (9.8%)	<0.001
A	1,068 (25.4%)	180 (16.8%)	403 (24.0%)	485 (33.4%)	
B	479 (11.4%)	91 (8.5%)	153 (9.1%)	235 (16.2%)	
C	2,282 (54.3%)	724 (67.5%)	989 (58.9%)	569 (39.2%)	
D	114 (2.7%)	48 (4.5%)	46 (2.7%)	20 (1.4%)	
**Tumor type, n (%)**					
Well-defined	3,038 (70.8%)	765 (66.1%)	1,134 (67.6%)	1,139 (78.2%)	<0.001
Poorly defined	1,253 (29.2%)	392 (33.9%)	544 (32.4%)	317 (21.8%)	
**Tumor number, n (%)**					
1	2,069 (48.2%)	506 (43.7%)	832 (49.6%)	731 (50.2%)	<0.001
2–3	956 (22.3%)	321 (27.7%)	385 (22.9%)	250 (17.2%)	
≥4	1,266 (29.5%)	330 (28.5%)	461 (27.5%)	475 (32.6%)	
**Tumor size (cm), n (%)**					
<2	609 (14.2%)	134 (11.6%)	230 (13.7%)	245 (16.8%)	<0.001
≥2, <5	1,653 (38.5%)	429 (37.1%)	598 (35.6%)	626 (43.0%)	
≥5, <10	1,147 (26.7%)	359 (31.0%)	439 (26.2%)	349 (24.0%)	
≥10	882 (20.6%)	235 (20.3%)	411 (24.5%)	236 (16.2%)	
**Portal vein thrombosis, n (%)**					
None	2,996 (69.8%)	782 (67.6%)	1,112 (66.3%)	1,102 (75.7%)	<0.001
1st or 2nd branch	873 (20.3%)	236 (20.4%)	401 (23.9%)	236 (16.2%)	
Main branch	422 (9.8%)	139 (12.0%)	165 (9.8%)	119 (8.1%)	
**Extrahepatic spread, n (%)**					
Negative	3,686 (85.9%)	939 (81.2%)	1,383 (82.4%)	1,364 (93.7%)	<0.001
Positive	605 (14.1%)	218 (18.8%)	295 (17.6%)	92 (6.3%)	
**MELD score** ^†^ **, n (%)**					
<10	2,724 (65.2%)	693 (64.8%)	1,024 (61.0%)	1,007 (70.5%)	<0.001
≥10, <20	1,345 (32.2%)	341 (31.9%)	607 (36.2%)	397 (27.8%)	
≥20, <30	92 (2.2%)	29 (2.7%)	41 (2.4%)	22 (1.5%)	
≥30	16 (0.4%)	7 (0.7%)	6 (0.4%)	3 (0.2%)	
**AFP**^‡^ **(ng/mL), n (%)**					
<20	1,336 (31.4%)	307 (26.6%)	488 (29.2%)	541 (37.9%)	<0.001
≥20, <200	966 (22.7%)	258 (22.4%)	394 (23.6%)	314 (22.0%)	
≥200	1,950 (45.9%)	587 (51.0%)	791 (47.3%)	572 (40.1%)	
**PIVKA-II**^¶^ **(mAU/mL), n (%)**					
<40	512 (29.8%)	11 (33.3%)	205 (27.4%)	296 (31.6%)	0.154
≥40	1,207 (70.2%)	22 (66.7%)	544 (72.6%)	641 (68.4%)	
**Initial treatment, n (%)**					
Liver transplantation	89 (2.1%)	0 (0.0%)	28 (1.7%)	61 (4.2%)	<0.001
RFA	191 (4.5%)	14 (1.2%)	67 (4.0%)	110 (7.6%)	
Resection	826 (19.2%)	148 (12.8%)	332 (19.8%)	346 (23.8%)	
cTACE	2,362 (55.0%)	707 (61.1%)	969 (57.7%)	686 (47.1%)	
Radiation therapy	106 (2.5%)	19 (1.6%)	54 (3.2%)	33 (2.3%)	
Cytotoxic chemotherapy	144 (3.4%)	36 (3.1%)	72 (4.3%)	36 (2.5%)	
Sorafenib	192 (4.5%)	0 (0.0%)	32 (1.9%)	160 (11.0%)	
Conservative treatment	351 (8.2%)	227 (19.6%)	106 (6.3%)	18 (1.2%)	
**Median follow-up duration, months (range)**	20.6 (0.1–192.0)	14.9 (0.1–192.0)	21.6 (0.1–146.5)	23.0 (0.2–86.2)	<0.001^††^

*Available in 4,201 patients

†Available in 4,177 patients

‡Available in 4,252 patients

¶Available in 1,719 patients

§Chi-square test unless otherwise specified

**ANOVA

††Kruskal-Wallis test

Abbreviations: HBV, hepatitis B virus; HCV, hepatitis C virus; IFN, interferon; DAA, direct-acting antiviral; SVR, sustained virologic response; ECOG PS, eastern cooperative oncology group performance status; UICC, union for international cancer control; BCLC, Barcelona clinic liver cancer; MELD, model for end-stage liver disease; AFP, alpha fetoprotein; PIVKA-II, protein induced by vitamin K absence/antagonist-II; RFA, radiofrequency ablation; cTACE, conventional trans-arterial chemoembolization.

### Performance status, liver function, and tumor stage

Chronologically, patients were diagnosed as HCC with better performance status and better liver function in the latter cohort: ECOG PS 0 in 33.3%, 48.6%, and 77.5% (*p*<0.001) and Child–Pugh class A in 74.6%, 79.9%, and 87.4% (*p*<0.001), respectively in cohort A to C (S1 Fig). In addition, the proportion of patients with a MELD score <10 was higher in cohort C than in cohort A (70.5% *vs*. 64.8%; *p*<0.001).

Patients were more likely to be diagnosed at an earlier stage in the latter cohort than in the early cohort. Only 8.0% of the patients were diagnosed with mUICC stage I disease in cohort A; this proportion was significantly increased to 12.0% in cohort C (*p*<0.001; S1 Fig). A similar finding was noted when the BCLC staging system was used. From cohort A to C, the proportion of patients with BCLC stage 0 increased from 2.7% to 9.8%, while stages C and D decreased from 72% to 40.6%. The baseline serum AFP level of cohort C was also significantly lower than that of cohort A.

### Initial treatment modality

In the entire cohort, conventional transarterial chemoembolization (cTACE) was the most frequent initial treatment modality (Table 1). However, the proportion of patients who were initially treated with TACE chronologically decreased; in contrast, the proportion of HCC patients who underwent surgical resection or RFA as the initial treatment modality significantly increased from. Upon initiation of LT in 2005 in the NCC, Korea, the proportion of patients who underwent LT increased from 1.7% in cohort B to 4.2% in cohort C. After the introduction of sorafenib for advanced HCC in 2008, the proportion of patients who were initially treated with sorafenib also increased from 1.9% to 11.0%, while that of patients managed with cytotoxic chemotherapy decreased from 4.3% to 2.5% in cohorts B and C, respectively.

### Survival analysis

**Fig 2:**
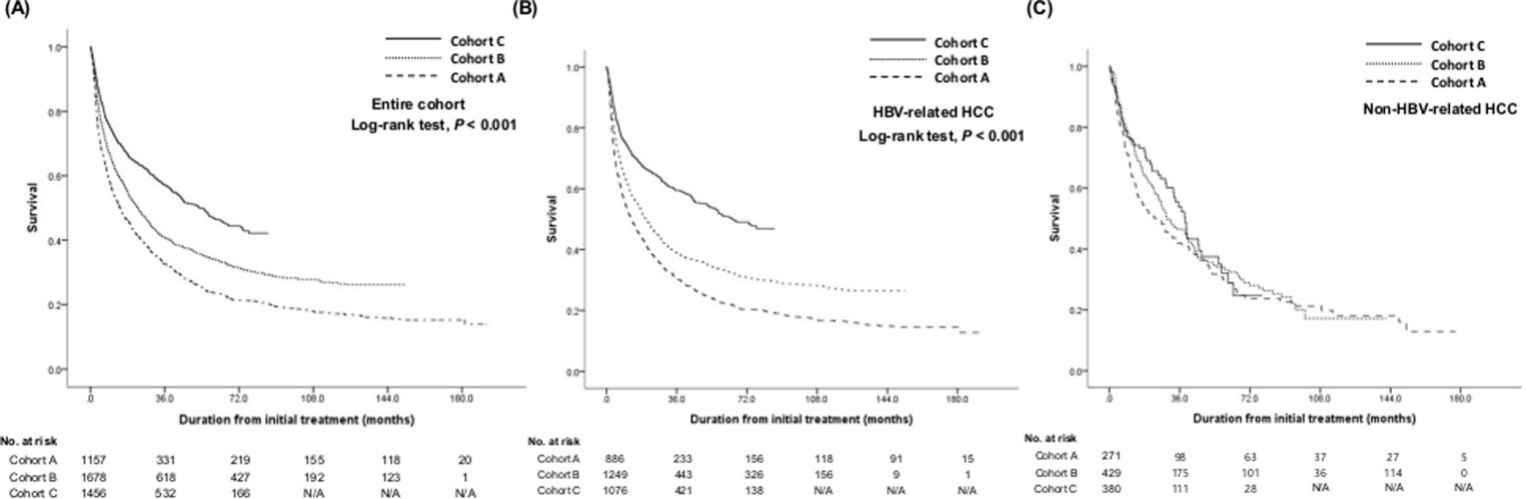
Survival of patients according to the subgroups in entire cohort (A), in patients with HBV etiology (B), and non-HBV etiology (C).

During a median 20.6 months follow-up period (range, 0.1–192.0 months), 2,782 patients (64.8%) died in the entire cohort. When chronologically analyzed, 82.8% of patients in cohort A, 70.0% in cohort B, and 44.4% in cohort C died until February 2017. The OS of the entire cohort and each sub-cohort is shown in S1 Table with stratified survival analysis. OS significantly increased in the latter cohort when adjacent cohorts were compared to each other (*p*<0.001 between cohorts A and B; *p*<0.001 between cohorts B and C; Fig 2). The 3-year OS rate and the median survival time significantly improved from cohort A, B, to C with 32.6%, 14.4 months (95% CI, 12.0–16.8 months) to 40.8%, 22.9 months (95% CI, 20.3–25.5 months), and to 57.0%, 53.6 months (95% CI, 45.7–61.5 months). In the stratified analysis according to age, sex, performance status, Child–Pugh class, mUICC stage, survival improvement was noted according to time. However, survival improvement was not definite in patients with etiology other than HBV, while patients with HBV-related HCC showed significant improvement in OS.

### Factors associated with overall survival

Using the Cox proportional hazard model, factors that were significantly associated with OS in the entire cohort were examined (Table 2). On univariate analysis, female sex, better ECOG PS, preserved liver function, early-stage HCC, favorable tumor characteristics (smaller size, fewer number of nodules, without portal vein invasion or extrahepatic metastasis), and lower tumor markers were associated with a lower risk of all-cause mortality. The multivariate analysis revealed better OS in female patients, patients with better ECOG PS, lower Child–Pugh class, lower mUICC stage, well-defined tumor, and lower AFP. In the entire cohort, the etiology of HCC was not a significant factor for OS. However, in cohort C, patients with HBV-related HCC showed significantly better OS than those with alcohol (hazard ratio [HR], 1.70; 95% CI, 1.31–2.21) and other etiologies (HR, 1.48; 95% CI, 1.1–1.98) (S2 Table).

**Table 2:** Factors associated with overall survival in entire cohort.

		Univariate analysis HR (95% CI)	*p*	Multivariate analysis HR (95% CI)	*p-value*
**Age**					
<50, n (%)	1,036 (24.1%)	1		1	
≥50, n (%)	3,255 (75.9%)	0.91 (0.83–0.99)	0.031	1.08 (0.98–1.18)	0.113
**Sex**					
Male	3,502 (81.6%)	1		1	
Female	789 (18.4%)	0.74 (0.67–0.82)	<0.001	0.83 (0.75–0.93)	0.001
**Cohort**					
A (2000–2004)	1,157 (27.0%)	1		1	
B (2005–2009)	1,678 (39.1%)	0.76 (0.69–0.82)	<0.001	0.94 (0.86–1.03)	0.175
C (2010–2015)	1,456 (33.9%)	0.52 (0.47–0.57)	<0.001	0.84 (0.75–0.95)	0.006
**Etiology**					
HBV	3,211 (74.8%)	1		1	
HCV	375 (8.7%)	0.98 (0.86–1.12)	0.775	1.00 (0.87–1.14)	0.945
Alcohol	318 (7.4%)	0.92 (0.80–1.06)	0.254	0.99 (0.85–1.15)	0.869
HBV+HCV	18 (0.4%)	1.14 (0.64–2.00)	0.661	1.61 (0.88–2.93)	0.12
Other	368 (8.6%)	1.00 (0.88–1.15)	0.973	0.98 (0.85–1.12)	0.72
**ECOG PS**					
0	2,230 (54.3%)	1		1	
1	1,864 (43.4%)	2.47 (2.29–2.67)	<0.001	1.41 (1.29–1.54)	<0.001
2	88 (2.1%)	3.29 (2.60–4.16)	<0.001	1.78 (1.40–2.27)	<0.001
	9 (0.2%)	4.63 (2.20–9.74)	<0.001	2.27 (1.07–4.79)	0.032
**Child–Pugh Class** ^*^					
A	3,408 (81.1%)	1		1	
B	687 (16.4%)	2.25 (2.05–2.47)	<0.001	1.56 (1.41–1.73)	<0.001
C	106 (2.5%)	2.34 (1.88–2.93)	<0.001	2.26 (1.78–2.85)	<0.001
**mUICC stage**					
I	410 (9.6%)	1		1	
II	1,304 (30.4%)	1.61 (1.31–1.97)	<0.001	1.66 (1.33–2.05)	<0.001
III	1,295 (30.2%)	4.46 (3.67–5.42)	<0.001	2.85 (2.31–3.51)	<0.001
IVa	828 (19.3%)	11.94 (9.78–14.58)	<0.001	4.66 (3.73–5.84)	<0.001
IVb	454 (10.6%)	14.56 (11.81–17.96)	<0.001	4.51 (3.56–5.72)	<0.001
**BCLC stage** ^*^					
0	258 (6.1%)	1			
A	1,068 (25.4%)	1.87 (1.40–2.49)	<0.001		
B	479 (11.4%)	4.52 (3.38–6.06)	<0.001		
C	2,282 (54.3%)	4.35 (6.36–10.98)	<0.001		
D	114 (2.7%)	9.30 (6.60–13.09)	<0.001		
**MELD score** ^**†**^					
<10	2,724 (65.2%)	1			
≥10, <20	1,345 (32.2%)	1.60 (1.48–1.73)	<0.001		
≥20, <30	92 (2.2%)	2.12 (1.66–2.70)	<0.001		
≥30	16 (0.4%)	1.86 (1.03–3.36)	0.041		
**Tumor number**					
1	2,069 (48.2%)	1			
2–3	956 (22.3%)	1.66 (1.50–1.83)	<0.001		
≥4	1,266 (29.5%)	3.64 (3.34–3.98)	<0.001		
**Tumor size (cm)**					
<2	609 (14.2%)	1			
≥2, <5	1,653 (38.5%)	1.73 (1.49–2.00)	<0.001		
≥5, <10	1,147 (26.7%)	3.89 (3.35–4.50)	<0.001		
≥10	882 (20.6%)	7.44 (6.40–8.65)	<0.001		
**Tumor type**					
Well-defined	3,038 (70.8%)	1		1	
Poorly defined	1,253 (29.2%)	4.19 (3.88–4.54)	<0.001	1.63 (1.48–1.79)	<0.001
**Portal vein thrombosis**					
None	2,996 (69.8%)	1			
1st or 2nd branch	873 (20.3%)	4.575 (4.19–5.00)	<0.001		
Main branch	422 (9.8%)	8.178 (7.28–9.18)	<0.001		
**Extrahepatic spread**					
Negative	3,686 (85.9%)	1			
Positive	605 (14.1%)	4.14 (3.77–4.56)	<0.001		
**AFP**^**‡**^ **(ng/ml)**					
<20	1,336 (31.4%)	1		1	
≥20, <200	966 (22.7%)	1.53 (1.37–1.71)	<0.001	1.17 (1.05–1.32)	0.007
≥200	1,950 (45.9%)	2.69 (2.45–2.95)	<0.001	1.58 (1.43–1.75)	<0.001
**PIVKA-II (mAU/ml)**					
<40	512 (29.8%)	1			
≥40	1,207 (70.2%)	3.01 (2.53–3.58)	<0.001		
**Initial treatment**					
Conservative treatment	351 (8.2%)	1		1	
Liver transplantation	89 (2.1%)	0.03 (0.02–0.04)	<0.001	0.06 (0.04–0.11)	<0.001
RFA	191 (4.5%)	0.04 (0.03–0.05)	<0.001	0.19 (0.14–0.27)	<0.001
Resection	826 (19.2%)	0.04 (0.03–0.05)	<0.001	0.14 (0.11–0.17)	<0.001
cTACE	2,362 (55.0%)	0.15 (0.14–0.17)	<0.001	0.36 (0.31–0.41)	<0.001
Radiation therapy	106 (2.5%)	0.35 (0.28–0.44)	<0.001	0.44 (0.34–0.56)	<0.001
Cytotoxic chemotherapy	144 (3.4%)	0.58 (0.47–0.71)	<0.001	0.68 (0.55–0.85)	0.001
Sorafenib	192 (4.5%)	0.73 (0.61–0.87)	<0.001	0.84 (0.68–1.04)	0.104
Other	30 (0.7%)	0.67 (0.46–0.98)	0.038	0.63 (0.43–0.93)	0.021

*Available from 4,201 patients, †Available in 4,177 patients, ‡Available in 4,252 patients, ¶Available from 1,719 patients

Abbreviations: HBV, hepatitis B virus; HCV, hepatitis C virus; ECOG PS, Eastern Cooperative Oncology Group Performance Status; UICC, Union for International Cancer Control; BCLC, Barcelona Clinic Liver Cancer; MELD, model for end-stage liver disease; AFP, alpha-fetoprotein; PIVKA-II, protein induced by vitamin K antagonist-II; RFA, radiofrequency ablation; cTACE, conventional transarterial chemoembolization.

Patients who underwent LT, surgical resection, or RFA as initial treatment showed the most significant mortality reduction, compared to those managed with conservative treatment (S2 Table). The use of sorafenib reduced HR for mortality in the univariate analysis; however, it lost significance in the multivariate model in the entire cohort. However, among cohort C, sorafenib significantly reduced the risk of mortality compared to conservative treatment (HR 0.05, 95% CI 0.03–0.11) (S2 Table).

### Survival difference in patients with HBV-related HCC and non-HBV-related HCC

In cohort C, we found that patients with HBV-related HCC showed better survival than those with alcohol or other etiologies. Moreover, among patients with HBV-related HCC, significant reduction in risk of OS was noted in the cohort B (HR 0.91, 95% CI 0.82–1.01) and C (HR 0.72, 95% CI 0.62–0.83) when compared to cohort A.

In the stratified analysis of patients with HBV-related HCC, chronological differences in mUICC stage at the time of diagnosis were noted (S4 Table). A higher proportion of patients were diagnosed with mUICC stage I and II (7.7% and 22.9% in cohort A *vs*. 8.9% and 28.7% in cohort B *vs*. 12.9% and 35.4% in cohort C; *p*<0.001). The stage at the time of diagnosis in patients with HCV- and alcohol-related HCC did not differ between cohorts A and C (*p* = 0.366 and *p* = 0.51, respectively; S3 Table).

**Fig 3:**
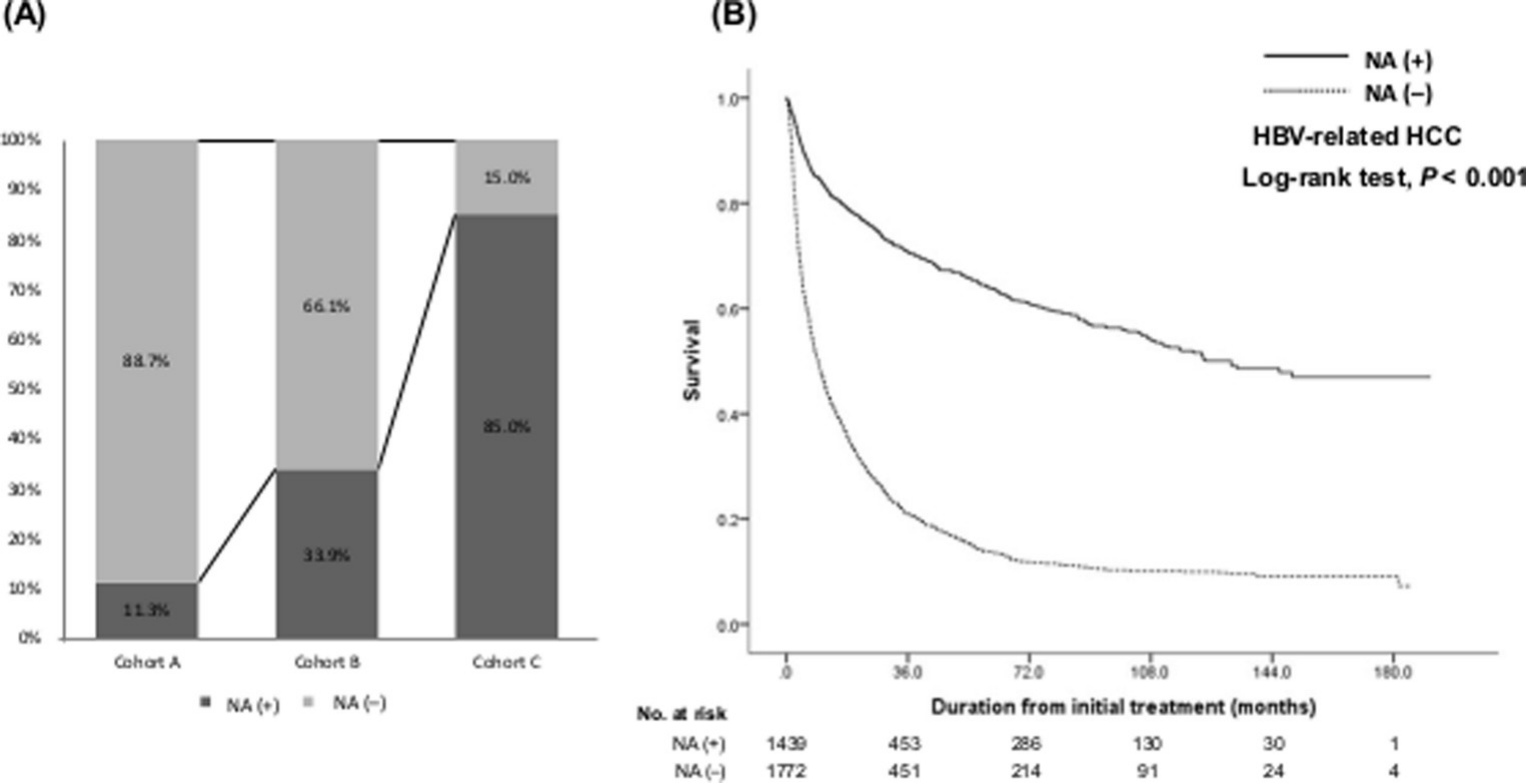
Proportion of patients who used nucleos(t)ide analogues (A) and the survival of patients according to the use of nucleos(t)ide analogues (B).

Nucleos(t)ide analogues (NAs) were used in 44.8% of HBV-related HCC patients during the study period. Only 11.3% of patients used NAs in cohort A, while 33.9% and 85.0% of patients in cohorts B and C used NAs, respectively (*p*<0.001; S4 Table, Fig 3A). The types of NAs and the number of patients treated with respective NAs are described in Table 1. In cohort C, most patients received anti-potent NAs including entecavir (47.0%) or tenofovir disoproxil fumarate (44.8%). Only 5 patients among HBV-related HCC patients in cohort A experienced interferon (IFN) therapy. When the OS of patients with HBV-related HCC was plotted according to the use of NAs, significant improvement was noted in patients who used NAs compared to those who did not (Fig 3B; adjusted HR 0.72, 95% CI 0.64–0.80) after adjusting for age, AFP, ECOG PS, CP class, and mUICC stage. Most patients who used NAs in cohort A had an earlier stage of HCC (stage I, 22.0%; stage II, 58.0%), while a higher proportion of patients who received NAs were diagnosed at an advanced stage in cohort B or C (S5 Table).

Among HCV-related HCC patients, only 18.4% had received anti-HCV treatment including IFN-based regimen (71.0%) and direct-acting antivirals (DAAs, 29.0%) (Table 1). DAAs (n = 20) included daclatasvir/asunaprevir (n = 12), glecaprevir/pibrentasvir (n = 2), sofosbuvir/ledipasvir (n = 3), sofosbuvir (n = 1), and elvascvir/grazoprevir (n = 1). Chronologically, more patients had received anti-HCV treatment over time. Also, significantly higher sustained virologic response (SVR) rate was also noted probably due to use of DAAs in cohort C. However, neither the use of anti-HCV treatment (HR 1.09; 95% CI 0.81–1.48; *p* = 0.56) nor the presence of SVR (HR 0.88; 95% CI 0.51–1.52; *p* = 0.643) were not significant factor for OS among HCV-related HCC patients.

## Discussion

This prospective cohort study showed significantly improved survival of patients with HCC in the HBV-endemic area. On comparing the three chronological prospective HCC cohorts for 16 years, significant changes in baseline performance status, stage of HCC, and underlying liver function were noted, which translated into improved survival in the latter cohort (cohort C > B > A). In stratified analysis, this improvement was mainly caused by improved survival in patients with HBV-related HCC rather than in those with non-HBV-related HCC. In South Korea, where HBV infection is endemic and accounts for the majority of *de novo* HCC, the baseline characteristics and clinical outcomes are different from those of western countries where HCV and alcohol are the major causes of HCC. This study might be of great value in understanding these differences in different populations.

The annual incidence of HCC is decreasing, and the 5-year survival rate of HCC patients has improved, reaching 32.8% according to a report from the National Cancer Registry in South Korea [3]. However, this study demonstrated that the 5-year survival rate was 47.0% in our cohort between 2010 and 2015. This higher survival rate reported from our institute may partly be explained by a specialized multidisciplinary approach in managing HCC to maximize the benefit from various treatments in NCC, Korea. Approximately 24% of patients were diagnosed at a younger age (<50 years) compared to data from western countries [19], which reflects the predominant viral etiology in the Korean HCC cohort. However, a subtle but constant increase in the age at the time of diagnosis was noted with a decrease in the proportion of HBV- or HCV-related HCC and an increase in the proportion of alcohol- or nonalcoholic steatohepatitis-related HCC during the study period. In cohort C, the majority of HCC patients were diagnosed without any significant symptoms and with preserved liver function. This may be partly explained by the role of NAs in preserving liver function in patients with HBV infection and partly by the utilization of surveillance programs, which may facilitate early detection in asymptomatic patients.

Efforts have been made to reduce the mortality of patients with HCC. The clinical outcome of patients with HCC is mainly decided by underlying liver function and tumor characteristics [20]. In hepatitis virus endemic areas, controlling underlying viral hepatitis may be a plausible approach to improve the survival of patients with HCC by preserving or reversing the deteriorated liver function [21]. A study from Taiwan showed that national immunization program lowered the incidence of HCC and the mortality for chronic liver disease and HCC [22]. Another study from Taiwan showed that the treatment with antiviral agent also decreased the incidence and mortality of HCC patients [23]. The use of NAs after curative resection for HBV-related HCC is reportedly associated with decreased tumor recurrence, possibly improving the survival [24, 25]. Thus, the current study also shows a significant result that the survival improvement in cohort C was mainly caused by improved survival in patients with HBV-related HCC with the use of NAs. In National Health Insurance System of South Korea, NAs can be used for chronic HBV patients without any restrictions on the type of drug and treatment duration since 2010 [19]. Moreover, improved treatment responses with the advent of potent antiviral agents (e.g. entecavir or tenofovir) might have substantially reduced the risk of HBV-related HCC [10, 26, 27]. Approximately 90% of patients with HBV-related HCC in cohort C had been treated with entecavir or tenofovir which are potent drugs that have shown superior virological and biochemical benefits as compared to lamivudine [28–32]. A higher potency antiviral agent might be better than a less potent drug for preventing hepatic decompensation, which may translate into a lower risk of HCC development and a better OS.

Another approach to improve the survival of HCC patients is to detect the disease at an early stage to facilitate the use of curative treatment. A previous study has shown that the surveillance program for HCC may be effective in mortality reduction [33]. Korea NCSP has been providing screening for liver cancer in high-risk populations aged ≥40 years since 2003. This program utilizes abdominal ultrasonography with serum AFP performed every 6 months. Even though the lifetime screening rate reached 54.3% in 2011, the screening rate according to the recommendation is still low at 22.9% in screening candidates [34]. Data regarding whether HCC was detected through screening or by incidence is not available in the current study to address this issue. Further studies are needed to evaluate whether the current screening program has actually led to survival improvement in patients with HCC.

In patients with non-HBV etiology, the mUICC stage at the time of diagnosis was similar between the groups, unlike the findings from patients with HBV etiology. In addition, the survival of patients did not differ significantly between cohort groups. This may partly be due to the public unawareness of the risk factors of HCC in those without HBV infection and the public environment that is usually generous of alcohol intake and morbid obesity in South Korea. This may also be explained by the lack of effective tools to improve liver function in these patients. Direct acting agents that can eliminate HCV infection have been developed only in recent years. Further survival improvement is anticipated in patients with HCV-related HCC.

This study has some limitations. Because the history of alcohol intake was based on the patient’s report, it was considered unreliable. Thus, if the patient had hepatitis virus infection with alcohol history at the same time, patients were considered to have a viral etiology rather than alcohol. This process may have led to a selection bias. In addition, we could not investigate the cause of death in patients who died during the study period. As a result, we were unable to present liver-related mortality in the current analysis. In the current study, survival benefit from sorafenib was recognized in mUICC stage IV patients among cohort C compared to conservative treatment. This study analyzed HCC patients diagnosed between 2000 and 2015, therefore we could not evaluate the efficacy of novel therapeutic modalities. In future cohort study, we will further evaluate the survival benefit from the new therapeutic modalities including combination therapy and novel systemic agents such as immune checkpoint inhibitors. Finally, although this is a prospective cohort study, the results of this study may not represent the general characteristics and outcomes of patients with HCC in Korea since our institute is a tertiary referral center.

## Conclusions

In summary, this prospective cohort study demonstrated the chronologically improved OS in HCC patients in Korea. This finding was mostly due to the improved survival of patients with HBV-related HCC. The use of NAs and the surveillance program for HCC high-risk population were significantly associated with improved survival in these patients, leading to the increased proportion of HCC patients with good liver function and small tumors. In addition, the use of novel systemic agents such as sorafenib might also improve the survival of patients with advanced-stage HCC. This study might be valuable in understanding the changes in baseline characteristics and clinical outcomes of HCC in HBV-endemic areas and building strategies to further improve the clinical outcomes of HCC patients.

## Supporting information

S1 Fig Chronologic change in (A) etiology, (B) Child–Pugh class, and (C) mUICC stage. (TIF)

S1 Table Stratified survival analysis in the chronological cohorts. (PDF)

S2 Table Multivariate analysis for overall survival in cohort C. (PDF)

S3 Table mUICC stage in patients with non-HBV etiology. (PDF)

S4 Table Overall survival in patients with HBV-related HCC. (PDF)

S5 Table Patients with HBV etiology who used NAs. (PDF)

## Abbreviations

HCC: hepatocellular carcinoma
HBV: hepatitis B virus
OS: overall survival
CI: confidence interval
NCSP: National Cancer Screening Program
NCC: National Cancer Center
PS: performance status
ECOG: Eastern Cooperative Oncology Group
AFP: alpha-fetoprotein
PIVKA-II: protein induced by vitamin K antagonist-II
mUICC: modified Union for International Cancer Control
BCLC: Barcelona Clinic for Liver Cancer
MELD: model for end-stage liver disease
CT: computed tomography
MRI: magnetic resonance imaging
LT: liver transplantation
RFA: radiofrequency ablation
TACE: transarterial chemoembolization
RT: radiation treatment
SD: standard deviation
HCV: hepatitis C virus
HR: hazard ratio
NAs: nucleos(t)ide analogues
IFN: interferon
DAA: direct-acting antiviral
SVR: sustained virologic response
