# Supplementary figures and images for: Better survival of patients with hepatitis B virus-related hepatocellular carcinoma in South Korea: Changes in 16-years cohorts
Source: PLoS One. 2022 Mar 24;17(3):e0265668. doi: 10.1371/journal.pone.0265668 (PMC8947113; doi:10.1371/journal.pone.0265668)

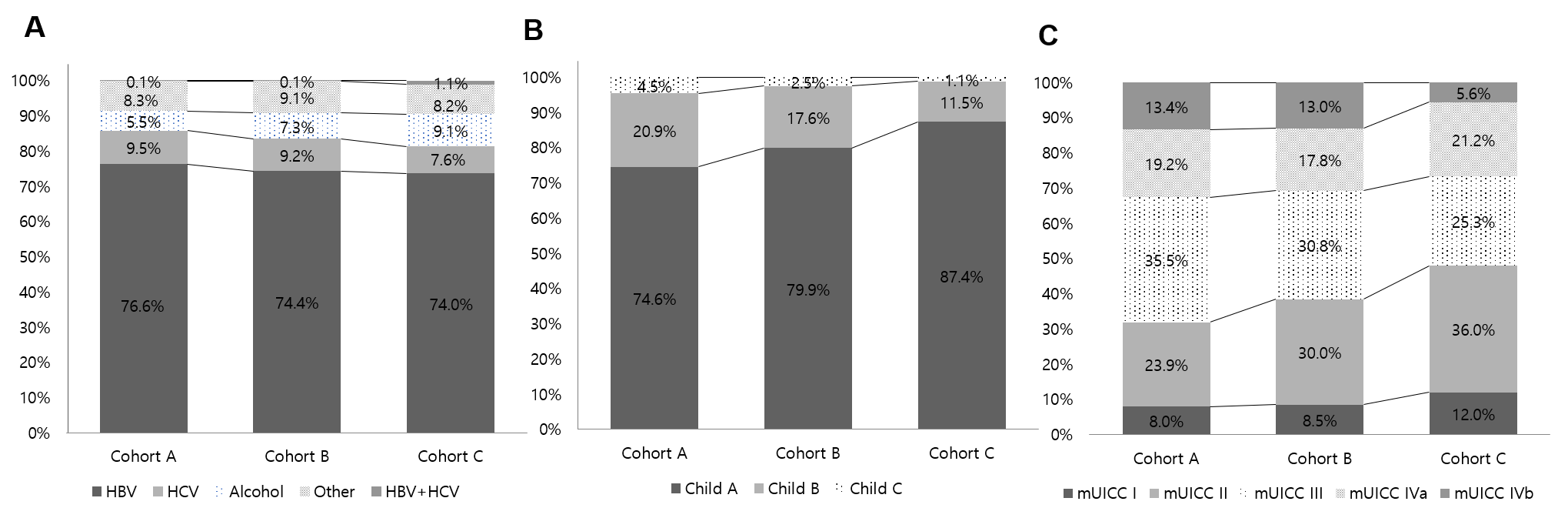

Supplement: S1 Fig — (TIF) [file pone.0265668.s001.tif]
