# Supplementary material for: Better survival of patients with hepatitis B virus-related hepatocellular carcinoma in South Korea: Changes in 16-years cohorts
Source: PLoS One. 2022 Mar 24;17(3):e0265668. doi: 10.1371/journal.pone.0265668 (PMC8947113; doi:10.1371/journal.pone.0265668)
Supplement: S1 Table — (PDF) [file pone.0265668.s002.pdf]

S1 Table. Stratified survival analysis in the chronological cohorts.

	Cohort A (n=1,157)					Cohort B (n=1,678)					Cohort C (n=1,456)					*p-value*‡
	n (%)	1-YSR	3-YSR	5-YSR	Median, (Mo, 95% CI)	n (%)	1-YSR	3-YSR	5-YSR	Median, (Mo, 95% CI)	n (%)	1-YSR	3-YSR	5-YSR	Median, (Mo, 95% CI)	
**Overall**	1,157	53.4	32.6	23.6	14.4 (12.0-16.8)	1,678	62.1	40.8	23.1	22.9 (20.3-25.5)	1,456	72.1	57.0	47.0	53.6 (45.7-61.5)	<0.001
**Age**																
<50	309 (26.7%)	43.7	29.1	24.4	8.1 (5.8-10.4)	443 (26.4%)	56.1	38.5	34.8	18.1 (12.6-23.6)	284 (19.5%)	66.4	53.0	46.1	50.5 (26.4-74.6)	<0.001
≥50	848 (73.3%)	56.9	33.9	23.3	17.6 (14.6-20.6)	1,235 (73.6%)	64.3	41.7	33.7	24.3 (21.1-27.5)	1,172 (80.5%)	73.5	58.0	47.2	55.4 (47.5-63.3)	<0.001
**Sex**																
Male	932 (80.6%)	52.0	31.9	22.8	13.2 (11.0-15.4)	1,398 (83.3%)	60.5	38.3	31.8	20.6 (18.1-23.1)	1,172 (80.5%)	70.1	54.5	44.2	44.6 (37.3-51.9)	<0.001
Female	225 (19.4%)	59.1	35.6	27.1	21.3 (15.0-27.6)	280 (16.7%)	69.8	53.5	45.3	41.9 (24.5-59.3)	284 (19.5%)	80.3	67.9	58.7	NR	<0.001
**Etiology**																
HBV	886 (76.6%)	51.2	30.2	22.4	12.7 (10.4-15.0)	1,249 (74.4%)	59.1	39.0	33.3	20.4 (17.5-23.3)	1,076 (74.0%)	72.5	59.3	51.4	64.5 (NA)	<0.001
HCV	110 (9.5%)	63.6	41.8	28.8	22.9 (10.0-35.8)	154 (9.2%)	74.4	46.5	33.1	29.3 (19.7-39.0)	111 (7.6%)	75.9	54.2	32.2	38.8 (33.4-44.2)	0.492
Alcohol	64 (5.5%)	65.6	45.2	30.6	30.3 (13.5-47.1)	122 (7.3%)	78.7	50.7	39.0	40.1 (25.2-55.0)	132 (9.1%)	69.5	49.5	29.5	35.6 (21.2-50.0)	0.216
Other	96 (8.3%)	53.1	35.4	24.0	14.6 (5.6-23.6)	152 (9.1%)	60.1	41.5	36.3	23.0 (12.4-33.6)	120 (8.2%)	69.7	48.1	40.6	33.0 (13.2-52.9)	0.021
**ECOG PS**																
0	385 (33.3%)	75.6	50.5	37.8	38.1 (31.3-44.9)	816 (48.6%)	79.0	57.5	50.2	61.1 (48.0-74.2)	1,129 (77.5%)	77.9	63.0	52.4	66.6 (NA)	<0.001
1	750 (64.8%)	42.3	23.5	16.3	8.0 (6.7-9.3)	821 (48.9%)	47.4	25.9	19.3	10.9 (8.9-12.9)	293 (20.1%)	52.9	36.9	28.3	15.0 (7.9-22.1)	<0.001
2	22 (1.9%)	40.9	27.3	22.7	5.0 (0-12.7)	38 (2.3%)	21.1	7.9	7.9	4.6 (0.7-8.5)	28 (1.9%)	41.8	33	0	9.4 (4.9-14.0)	0.064
3	0 (0.0%)	NA	NA	NA	NA	3 (0.2%)	0	0	0	0.9 (0-2.2)	6 (0.4%)	45.5	22.7	0	8.8 (0-18.2)	0.024

**Child–Pugh class***																
A	800 (74.6%)	62.1	41.1	30.0	23.3 (19.2-27.4)	1,340 (79.9%)	67.1	45.3	38.1	28.7 (25.0-32.4)	1,268 (87.4%)	75.5	59.7	49.3	57.5 (47.5-67.5)	<0.001
B	224 (22.9%)	31.1	10.8	6.2	4.7 (3.8-5.6)	296 (17.6%)	42.0	21.2	16.0	8.4 (6.3-10.5)	167 (11.5%)	47	36.8	28.1	10.9 (3.7-18.1)	<0.001
C	48 (4.5%)	13.7	2.3	0	1.3 (0.8-1.8)	42 (2.5%)	42.9	35.7	30.6	4 (0-10.8)	16 (1.1%)	74.2	74.2	74.2	NA	<0.001
**mUICC stage**																
I	92 (8.0%)	90.2	71.5	61.3	152.1 (NA)	143 (8.5%)	98.6	84.7	74.9	NR	175 (12.0%)	97.7	89.1	81.2	NR	0.004
II	277 (23.9%)	85.9	65.3	51.0	64.0 (46.4-81.6)	503 (30.0%)	86.6	71.1	62.2	111.4 (NA)	524 (36.0%)	91.8	81.9	69.1	NR	<0.001
III	411 (35.5%)	56.0	26.4	16.2	15.2 (12.0-18.4)	516 (30.8%)	65.1	34.2	26.1	21.6 (18.8-24.4)	368 (25.3%)	73.5	50.3	36.6	37.0 (27.9-46.1)	<0.001
IVa	222 (19.2%)	17.6	6.6	2.3	4.0 (3.5-4.5)	298 (17.8%)	26.4	6.7	4.2	5.1 (4.3-6.0)	308 (21.2%)	34.4	14.9	10.2	6.5 (5.3-7.7)	<0.001
IVb	155 (13.4%)	17.5	4	2	3.5 (2.8-4.2)	218 (13.0%)	23.9	5.4	2.4	4.2 (3.2-5.2)	81 (5.6%)	24.8	13.2	13.2	4.5 (2.9-6.1)	0.136
**BCLC stage***																
0	29 (2.7%)	96.6	86.2	72.4	123.4 (NA)	87 (5.2%)	98.8	90.4	81.8	NR	142 (9.8%)	99.3	91.6	84.0	NR	0.173
A	180 (16.8%)	88.3	64.4	50.4	61.2 (43.5-78.9)	403 (24.0%)	91.3	75.9	68.1	NR	485 (33.4%)	93.1	83.2	69.5	NR	<0.001
B	91 (8.5%)	64.8	34.8	17.7	20.3 (12.2-28.4)	153 (9.1%)	83.6	43.9	33.5	31.8 (27.3-36.3)	235 (16.2%)	77.0	50.9	43.0	38.4 (29.0-47.8)	0.001
C	724 (67.5%)	44.3	24.9	17.4	8.9 (7.5-10.3)	989 (58.9%)	44.6	22.0	16.1	9.9 (8.7-11.1)	569 (39.2%)	45.4	27.7	20.5	9.4 (7.4-11.4)	0.467
D	48 (4.5%)	13.7	2.3	0	1.3 (0.8-1.8)	46 (2.7%)	43.5	37.0	32.3	4.0 (0-10.9)	20 (1.4%)	69.2	63.5	63.5	NA	<0.001
**Tumor type**																
Well-defined	765 (66.1%)	71.4	45.4	33.3	30.1 (25.7-34.5)	1,134 (67.6%)	77.6	54.7	46.4	50.1 (41.3-58.9)	1,139 (78.2%)	83.9	68.0	56.7	NR	<0.001
Poorly defined	392 (33.9%)	18.2	7.4	4.5	3.7 (3.2-4.2)	544 (32.4%)	29.9	12.1	8.6	5.6 (4.8-6.4)	317 (21.8%)	29.3	16.8	10.7	5.7 (4.7-6.7)	<0.001
**Tumor**																

**number**																
1	506 (43.7%)	63.8	48.6	39.1	33.6 (24.8-42.4)	832 (49.6%)	71.2	56.0	49.1	57.5 (45.8-49.2)	731 (50.2%)	88.5	78.2	67.9	NR	<0.001
2-3	321 (27.7%)	58.9	29.0	18.9	17.2 (14.2-20.2)	385 (22.9%)	69.2	38.9	29.2	26.7 (22.5-31.0)	250 (17.2%)	84.6	64.9	48.2	57.5(44.0-71.0)	<0.001
≥4	330 (28.5%)	31.9	11.3	4.2	5.0 (3.7-6.3)	461 (27.5%)	39.7	15.0	10.7	7.1 (5.4-8.8)	475 (32.6%)	40.1	19.6	13.8	7.3 (5.9-8.7)	<0.001
**Tumor size (cm)**																
<2	134 (11.6%)	87.3	64.0	52.4	80.4 (31.0-130.0)	230 (13.7%)	94.3	72.1	61.3	NR	245 (16.8%)	95.4	86.0	75.2	NR	<0.001
≥2, <5	429 (37.1%)	74.6	43.7	31.3	30.3 (26.3-34.3)	598 (35.6%)	81.8	62.3	51.5	63.5 (52.8-74.2)	626 (43.0%)	84.2	69.1	55.2	74.1 (NA)	<0.001
≥5, <10	359 (31.0%)	38.2	21.9	14.5	7.6 (6.3-8.9)	439 (26.2%)	52.8	23.3	21.4	13.7 (10.7-16.7)	349 (24.0%)	59.8	40.2	34.5	18.0 (12.8-23.2)	<0.001
≥10	235 (20.3%)	18.4	10.5	7.0	3.1 (2.7-3.5)	411 (24.5%)	25.5	11.2	7.1	5.4 (4.5-6.3)	236 (16.2%)	33.5	18.4	12.9	6.4 (5.0-7.8)	<0.001
**Portal vein thrombosis**																
None	782 (67.6%)	72.9	45.5	33.3	30.3 (26.34.6)	1,112 (66.3%)	81.5	58.8	49.4	58.0 (48.9-67.1)	1,102 (75.7%)	85.2	70.1	58.4	NR	<0.001
1st or 2nd branch	236 (20.4%)	16.9	7.9	4.8	3.7 (3.0-4.4)	401 (23.9%)	27.0	6.8	5.0	5.8 (5.0-6.6)	236 (16.2%)	39.6	22.3	15.9	7.0 (5.4-8.6)	<0.001
Main branch	139 (12.0%)	1.9	1.1	0.8	2.8 (2.4-3.2)	165 (9.8%)	2.9	1.4	0.9	3.8 (3.2-4.4)	119 (8.1%)	3.2	1.7	1.3	3.8 (3.3-4.3)	0.004
**Extrahepatic spread**																
Negative	939 (81.2%)	61.7	39.0	28.6	22.3 (18.8-25.8)	1,383 (82.4%)	70.3	48.4	40.7	32.7 (27.8-37.6)	1,364 (93.7%)	75.0	59.9	49.2	57.3 (47.4-67.2)	<0.001
Positive	218 (18.8%)	17.5	4.7	1.9	3.6 (2.9-4.3)	295 (17.6%)	23.6	5.6	2.6	4.4 (3.5-5.3)	92 (6.3%)	28.8	13.6	13.6	4.6 (3.0-6.2)	0.041
**AFP† (ng/mL)**																
<20	307 (26.6%)	76.2	53.9	40.8	42.9 (33.8-52.0)	488 (29.2%)	83.1	61.6	52.5	66.3 (53.4-79.2)	541 (37.9%)	88.7	76.8	62.2	NR	<0.001
≥20, <200	258	65.5	36.3	24.9	23.4 (17.9-29.0)	394	73.2	45.0	34.7	29.6 (24.6-34.6)	314	78.4	59.2	48.4	57.3 (38.6-76.0)	<0.001

	(22.4%)					(23.6%)					(22.0%)					
≥200	587 (51.0%)	36.6	20.0	14.2	6.6 (5.4-7.8)	791 (47.3%)	43.7	25.9	22.2	8.7 (7.3-10.1)	572 (40.1%)	52.9	36.8	31.4	14.9 (11.3-18.5)	<0.001
**Initial treatment**																
Liver transplantation	0 (0.0%)	NA	NA	NA	NA	28 (1.7%)	96.4	82.1	82.1	NR	61 (4.2%)	91.7	84.3	80.3	NR	0.827
RFA	14 (1.2%)	71.4	57.1	42.9	37.6 (0-77.6)	67 (4.0%)	100	89.3	81.3	NR	110 (7.6%)	94.4	89.7	73.2	NR	0.008
Resection	148 (12.8%)	89.9	79.0	68.1	128.3 (94.2-162.4)	332 (19.8%)	92.7	79.5	74.0	NR	346 (23.8%)	94.4	88.6	82.6	NR	0.001
cTACE	707 (61.1%)	62.7	34.8	22.8	19.9 (16.8-23.0)	969 (57.7%)	62.5	34.2	25.0	19.8 (17.6-22.0)	686 (47.1%)	74.3	50.6	36.3	36.9 (31.6-42.2)	<0.001
Radiation therapy	19 (1.6%)	15.8	0	0	4.1 (3.1-5.1)	54 (3.2%)	33.3	5.3	2.6	8.1 (4.8-11.4)	33 (2.3%)	56.9	38.2	NA	23.6 (5.8-41.5)	<0.001
Cytotoxic chemotherapy	36 (3.1%)	15.5	0	0	3.6 (2.2-5.0)	72 (4.3%)	11.1	1.4	NA	3.6 (2.9-4.3)	36 (2.5%)	35.2	21.4	NA	6.8 (4.8-8.8)	0.005
Sorafenib	0 (0.0%)	NA	NA	NA	NA	32 (1.9%)	12.5	3.1	NA	2.8 (1.0-4.6)	160 (11.0%)	13.0	3.5	2.1	3.7 (3.2-4.2)	0.550
Conservative treatment	227 (19.6%)	9.9	2.3	0	2.7 (2.4-3.0)	106 (6.3%)	1.9	0	0	2.0 (1.7-2.3)	18 (1.2%)	0	0	0	0.4 (0-0.8)	<0.001

*Available in 4,201 patients, †Available in 4,252 patients, ‡Log-Rank test

Abbreviations: HBV, hepatitis B virus; HCV, hepatitis C virus; ECOG PS, Eastern Cooperative Oncology Group Performance Status; UICC, Union for International Cancer Control; BCLC, Barcelona Clinic Liver Cancer; AFP, alpha-fetoprotein; RFA, radiofrequency ablation; cTACE, conventional transarterial chemoembolization; YSR, year survival rate; Mo, months; CI, confidence interval; NA, not available; NR, not reached.
