# Supplementary material for: Better survival of patients with hepatitis B virus-related hepatocellular carcinoma in South Korea: Changes in 16-years cohorts
Source: PLoS One. 2022 Mar 24;17(3):e0265668. doi: 10.1371/journal.pone.0265668 (PMC8947113; doi:10.1371/journal.pone.0265668)
Supplement: S2 Table — (PDF) [file pone.0265668.s003.pdf]

S2 Table. Multivariate analysis for overall survival in cohort C

	n (%)	Multivariate (n=1,421) HR (95% CI)	*p-value*
**Age**			
<50	281 (19.8%)	1	
≥50	1,140 (81.2%)	1.08 (0.88-1.33)	0.473
**Sex**			
Male	1,146 (80.6%)	1	
Female	275 (19.4%)	0.99 (0.74-1.33)	0.941
**Etiology**			
HBV	1,052 (74.0%)	1	
HCV	108 (7.6%)	0.99 (0.74-1.33)	0.941
Alcohol	131 (9.2%)	1.70 (1.31-2.21)	<0.001
HBV+HCV	16 (1.1%)	1.70 (0.89-3.22)	0.106
Other	114 (8.0%)	1.48 (1.10-1.98)	0.009
**ECOG PS**			
0	1,102 (54.3%)	1	
1	288 (43.4%)	1.32 (1.10-1.59)	0.003
2	25 (2.1%)	1.38 (0.82-2.33)	0.219
3	6 (0.2%)	1.97 (0.73-5.36)	0.182
**Child-Pugh Class**			
A	1,243 (77.6%)	1	
B	162 (11.4%)	2.11 (1.69-2.63)	<0.001
C	16 (1.1%)	2.17 (0.41-3.34)	0.771
**mUICC stage**			
I	167 (11.8%)	1	
II	514 (36.2%)	1.98 (1.25-3.14)	0.004
III	361 (25.4%)	3.35 (2.12-5.31)	<0.001
IVa	300 (21.1%)	5.58 (3.44-9.04)	<0.001
IVb	79 (5.6%)	4.60 (2.67-7.90)	<0.001
**Tumor type**			
Well-defined	1,113 (78.3%)	1	
Poorly defined	308 (21.7%)	1.65 (1.33-2.04)	<0.001
**AFP‡ (ng/ml)**			
<20	538 (37.9%)	1	
≥20, <200	313 (22.0%)	1.24 (0.97-1.59)	<0.001
≥200	570 (40.1%)	1.92 (1.55-2.38)	<0.001
**Initial treatment**			
Conservative treatment	16 (1.1%)	1	
Liver transplantation	58 (4.1%)	0.01 (0.00-0.01)	<0.001
RFA	107 (7.5%)	0.01 (0.00-0.3)	<0.001
Resection	340 (23.9%)	0.1 (0.0-0.01)	<0.001
cTACE	672 (47.3%)	0.02 (0.01-0.04)	<0.001
Radiation therapy	32 (2.3%)	0.02 (0.01-0.04)	<0.001
Cytotoxic chemotherapy	34 (2.4%)	0.05 (0.02-0.10)	<0.001
Sorafenib	156 (11.0%)	0.05 (0.03-0.11)	<0.001
Other	6 (0.4%)	0.08 (0.03-0.23)	<0.001

Abbreviations: HBV, hepatitis B virus; HCV, hepatitis C virus; ECOG PS, eastern cooperative oncology group performance status; UICC, union for international cancer control; AFP, alpha fetoprotein; RFA, radiofrequency ablation; cTACE, conventional trans-arterial chemoembolization.
