# Supplementary material for: Better survival of patients with hepatitis B virus-related hepatocellular carcinoma in South Korea: Changes in 16-years cohorts
Source: PLoS One. 2022 Mar 24;17(3):e0265668. doi: 10.1371/journal.pone.0265668 (PMC8947113; doi:10.1371/journal.pone.0265668)
Supplement: S3 Table — (PDF) [file pone.0265668.s004.pdf]

S3 Table. mUICC stage in patients with non-HBV etiology

**Etiology/ mUICC stage**	**Total**	**Cohort A**	**Cohort B**	**Cohort C**	***p-value***
**HCV**	n=375	n=110	n=154	n=111	
I	34 (9.1%)	10 (9.1%)	13 (8.4%)	11 (9.9%)	0.366
II	107 (28.5%)	27 (24.5%)	44 (28.6%)	36 (32.4%)	
III	144 (38.4%)	50 (45.5%)	60 (39.0%)	34 (30.6%)	
IVa	63 (16.8%)	18 (16.4%)	22 (14.3%)	23 (20.7%)	
IVb	27 (7.2%)	5 (4.5%)	15 (9.7%)	7 (6.3%)	
**Alcohol**	n=318	n=64	n=122	n=132	
I	34 (10.7%)	8 (12.5%)	12 (9.8%)	14 (10.6%)	0.351
II	126 (39.6%)	20 (31.3%)	51 (41.8%)	55 (41.7%)	
III	90 (28.3%)	22 (34.4%)	30 (24.6%)	38 (28.8%)	
IVa	49 (15.4%)	8 (12.5%)	19 (15.6%)	22 (16.7%)	
IVb	19 (6.0%)	6 (9.4%)	10 (8.2%)	3 (2.3%)	
**Other**	n=368	n=96	n=152	n=120	
I	23 (6.3%)	6 (6.3%)	7 (4.6%)	10 (8.3%)	0.371
II	119 (32.3%)	26 (27.1%)	49 (32.2%)	44 (36.7%)	
III	124 (33.7%	32 (33.3%)	58 (38.2%)	34 (28.3%)	
IVa	57 (15.5%)	18 (18.8%)	18 (11.8%)	21 (17.5%)	
IVb	45 (12.2%)	14 (14.6%)	20 (13.2%)	11 (9.2%)	
