# Supplementary material for: Better survival of patients with hepatitis B virus-related hepatocellular carcinoma in South Korea: Changes in 16-years cohorts
Source: PLoS One. 2022 Mar 24;17(3):e0265668. doi: 10.1371/journal.pone.0265668 (PMC8947113; doi:10.1371/journal.pone.0265668)
Supplement: S4 Table — (PDF) [file pone.0265668.s005.pdf]

S4 Table. Overall survival in patients with HBV-related HCC

	Cohort A (n=886)					Cohort B (n=1,249)					Cohort C (n=1,076)					*p*†	*p*‡
	n (%)	1-YSR	3-YSR	5-YSR	Median, (Mo, 95% CI)	n (%)	1-YSR	3-YSR	5-YSR	Median, (Mo, 95% CI)	n (%)	1-YSR	3-YSR	5-YSR	Median, (Mo, 95% CI)		
**Age**																	
<50	284 (32.1%)	44.0	29.2	24.1	8.2 (5.6-10.8)	409 (32.7%)	55.6	38.5	34.8	17.9 (12.1-23.8)	261 (24.3%)	66.5	52.7	47.0	13.9 (20.7-75.3)	<0.001	<0.001
≥50	602 (67.9%)	54.6	30.7	21.6	15.8 (12.4-19.2)	840 (67.3%)	60.9	39.3	32.6	21.2 (17.7-24.7)	815 (75.7%)	74.4	61.5	52.8	66.6 (NA)		<0.001
**Sex**																	
Male	712 (80.4%)	49.6	28.9	21.0	11.7 (9.4-14.0)	1,042 (83.4%)	57.5	36.2	30.8	18.0 (15.2-20.8)	859 (79.8%)	70.1	56.2	48.1	55.5 (42.3-68.7)	0.056	<0.001
Female	174 (19.6%)	57.5	35.6	28.1	21.3 (14.1-28.5)	207 (16.6%)	67.6	53.2	45.6	41.9 (19.0-64.8)	217 (20.2%)	82.4	72.2	64.6	NR		<0.001
**ECOG PS**																	
0	291 (32.8%)	72.9	47.9	37.4	34.7 (27.8-41.6)	603 (48.3%)	77.8	57.4	50.0	60.6 (43.7-77.5)	863 (80.2%)	78.3	65.3	57.2	NR	<0.001	<0.001
1	578 (65.2%)	40.7	21.5	15.0	7.3 (5.8-8.8)	620 (49.6%)	43.0	22.9	18.4	8.9 (7.3-10.5)	199 (18.5%)	49.7	35.5	26.6	11.7 (5.3-18.1)		0.001
2	17 (1.9%)	35.3	23.5	17.6	4.2 (1.4-7.0)	25 (2.0%)	16.0	4	NA	3.0 (0.9-5.1)	10 (0.9%)	36.8	N/A	N/A	7.0 (1.1-12.9)		0.055
**Child–Pugh class**[*]																	
A	608 (74.4%)	60.2	38.2	28.4	20.8 (16.7-24.9)	990 (79.3%)	64.2	43.8	37.6	24.4 (22.5-30.3)	932 (86.9%)	75.8	61.8	53.6	74.1 (NA)	<0.001	<0.001
B	175 (21.4%)	27.8	9.3	5.1	4.2 (3.1-5.3)	223 (17.9%)	40.2	19.3	15.3	7.4 (4.3-10.5)	125 (11.7%)	49.6	414	33.9	11.8 (0-27.1)		<0.001
C	34 (4.2%)	13.4	3.4	0	1.1 (0.4-1.8)	36 (2.9%)	38.9	30.6	27.5	3.5 (0.2-6.8)	15 (1.4%)	NA	NA	NA	NA		<0.001
**mUICC**																	

**stage**																	
I	68 (7.7%)	89.7	70.3	61.3	123.4 (NR)	111 (8.9%)	98.2	85.9	77.1	NR	139 (12.9%)	99.3	90.4	82.0	NR	<0.001	0.002
II	203 (22.9%)	84.2	62.6	49.5	59.9 (43.0-76.8)	358 (28.7%)	86.0	71.0	62.6	NR	381 (35.4%)	93.9	85.0	76.0	NR		<0.001
III	307 (34.7%)	55.7	25.2	15.8	15.2 (11.5-18.9)	368 (29.5%)	63.4	32.4	25.8	19.2 (16.0-22.4)	258 (24.0%)	75.6	55.4	43.5	52.3 (37.6-67.0)		<0.001
IVa	178 (20.1%)	16.3	5.4	2.3	3.9 (3.3-4.5)	239 (19.1%)	22.0	5.5	3.8	4.8 (4.0-5.6)	238 (22.1%)	31.9	15.3	11.7	6.1 (4.8-7.4)		<0.001
IVb	130 (14.7%)	16.3	4.1	2.4	3.1 (2.5-3.7)	173 (13.9%)	21.4	3.9	1.9	3.9 (3.3-4.5)	60 (5.6%)	20	NA	NA	3.8 (1.9-5.7)		0.381
**Use of nucleos(t)ide analogues, n (%)**																	
Yes	100 (11.3%)	100	100	99	NR	424 (33.9%)	96.0	84.3	76.1	NR	915 (85.0%)	74.3	60.5	53.0	74.1 (NA)	<0.001	<0.001
No	786 (88.7%)	45.0	21.3	12.5	9.5 (8.0-11.0)	825 (66.1%)	40.1	15.3	10.7	8.1 (7.1-9.1)	161 (15.0%)	62.2	52.9	42.7	48.0 (23.0-73.0)		<0.001

*Available from 3,138 patients, †Chi-square test, ‡Log-rank test.

Abbreviations: HBV, hepatitis B virus; ECOG PS, Eastern Cooperative Oncology Group Performance Status; UICC, Union for International Cancer Control; YSR, year survival rate; Mo, months; CI, confidence interval; NA, not available; NR, not reached.
