# Supplementary material for: Better survival of patients with hepatitis B virus-related hepatocellular carcinoma in South Korea: Changes in 16-years cohorts
Source: PLoS One. 2022 Mar 24;17(3):e0265668. doi: 10.1371/journal.pone.0265668 (PMC8947113; doi:10.1371/journal.pone.0265668)
Supplement: S5 Table — (PDF) [file pone.0265668.s006.pdf]

S5 Table. Patients with HBV etiology who used NAs

	**Total (n=1,439)**	**Cohort A (n=100)**	**Cohort B (n=424)**	**Cohort C (n=915)**	***p-value*** †
**Age**					
<50, n (%)	409 (28.4%)	40 (40.0%)	141 (33.3%)	228 (24.9%)	<0.001
≥50, n (%)	1030 (71.6%)	60 (60.0%)	283 (66.7%)	687 (75.1%)	
**Sex, n (%)**					
Male	1,143 (79.4%)	73 (73.0%)	333 (78.5%)	737 (80.5%)	0.18
Female	296 (20.6%)	27 (27.0%)	91 (21.5%)	78 (19.5%)	
**ECOG PS, n (%)**					
0	1,081 (75.1%)	51 (51.0%)	294 (69.3%)	736 (80.4%)	<0.001
1	341 (23.7%)	47 (47.0%)	127 (30.0%)	167 (18.3%)	
2	13 (0.9%)	2 (2.0%)	3 (0.7%)	8 (0.9%)	
3	4 (0.3%)	0 (0%)	0 (0%)	4 (0.4%)	
**Child-Pugh class*, n (%)**					
A	1,244 (87.1%)	89 (97.8%)	369 (87.0%)	786 (86.1%)	0.021
B	159 (11.1%)	2 (2.2%)	45 (10.6%)	112 (12.3%)	
C	25 (1.8%)	0 (0%)	10 (2.4%)	15 (1.6%)	
**mUICC stage, n (%)**					
I	227 (15.8%)	22 (22.0%)	78 (18.4%)	127 (13.9%)	<0.001
II	574 (39.9%)	58 (58.0%)	193 (45.5%)	323 (35.3%)	
III	356 (24.7%)	18 (18.0%)	117 (27.6%)	221 (24.2%)	
IVa	215 (14.9%)	1 (1.0%)	20 (4.7%)	194 (21.2%)	
IVb	67 (4.7%)	1 (1.0%)	16 (3.8%)	50 (5.5%)	

*Available in 1,439 patients, †Chi-square test unless otherwise specified

Abbreviations: HBV, hepatitis B virus; NAs, nucleos(t)ide analogues; ECOG PS, eastern cooperative oncology group performance status; UICC, union for international cancer control.
